# Characterization of Behavioral, Signaling and Cytokine Alterations in a Rat Neurodevelopmental Model for Schizophrenia, and Their Reversal by the 5-HT_6_ Receptor Antagonist SB-399885

**DOI:** 10.1007/s12035-018-0940-0

**Published:** 2018-02-08

**Authors:** Sinead E. Shortall, Ola H. Negm, Maxine Fowler, Lucy C. Fairclough, Patrick J. Tighe, Peter M. Wigmore, Madeleine V. King

**Affiliations:** 1 School of Life Sciences, Medical School, Queen’s Medical Centre, The University of Nottingham, Nottingham, NG7 2UH UK; 2 School of Medicine, Medical School, Queen’s Medical Centre, The University of Nottingham, Nottingham, NG7 2UH UK

**Keywords:** 5-HT_6_ receptor, Social isolation, Schizophrenia, Cytokines, Hippocampus, JNK mitogen-activated protein kinases

## Abstract

Post-weaning social isolation of rats produces neuroanatomical, neurochemical and behavioral alterations resembling some core features of schizophrenia. This study examined the ability of the 5-HT_6_ receptor antagonist SB-399885 to reverse isolation-induced cognitive deficits, then investigated alterations in hippocampal cell proliferation and hippocampal and frontal cortical expression of selected intracellular signaling molecules and cytokines. Male Lister hooded rats (weaned on post-natal days 21–24 and housed individually or in groups of 3–4) received six i.p. injections of vehicle (1% Tween 80, 1 mL/kg) or SB-399885 (5 or 10 mg/kg) over a 2-week period starting 40 days post-weaning, on the days that locomotor activity, novel object discrimination (NOD), pre-pulse inhibition of acoustic startle and acquisition, retention and extinction of a conditioned freezing response (CFR) were assessed. Tissue was collected 24 h after the final injection for immunohistochemistry, reverse-phase protein microarray and western blotting. Isolation rearing impaired NOD and cue-mediated CFR, decreased cell proliferation within the dentate gyrus, and elevated hippocampal TNFα levels and Cdc42 expression. SB-399885 reversed the NOD deficit and partially normalized CFR and cell proliferation. These effects were accompanied by altered expression of several members of the c-Jun N-terminal Kinase (JNK) and p38 MAPK signaling pathways (including TAK1, MKK4 and STAT3). Although JNK and p38 themselves were unaltered at this time point hippocampal TAK1 expression and phosphorylation correlated with visual recognition memory in the NOD task. Continued use of this neurodevelopmental model could further elucidate the neurobiology of schizophrenia and aid assessment of novel therapies for drug-resistant cognitive symptoms.

## Introduction

Schizophrenia has a complex etiology involving genetic and early-life environmental factors, leading to persistent neurodevelopmental changes [1]. Conservative estimates suggest the disease affects 5 million people in Europe alone with an annual cost of almost €94 billion [2]. It is characterized by positive, negative and cognitive symptoms and although antipsychotics are relatively effective in treating the former, negative and cognitive symptoms remain poorly managed and prevent reintegration into society [3]. There is consequently a significant demand for novel treatments for these symptom domains, and reliable predictive animal models are a tool to improve our understanding of their neurobiology and evaluation of potential therapeutics.

Social adversity is one environmental influence linked to human schizophrenia; for example individuals in urban areas with low social cohesion, or from ethnic minority groups show a higher incidence, as do those with hearing impairment (which may result in social isolation or defeat). Parental separation or loss and abuse or bullying during childhood, frequent relocation during adolescence, and social disadvantage or exclusion extending into later life have all been identified as risk factors [4]. Social deprivation can be replicated in rodents by post-weaning isolation of gregarious rat pups in individual cages. This neurodevelopmental manipulation produces lasting neuroanatomical, neurochemical and behavioral alterations which emerge post-puberty and resemble some of the core features of schizophrenia [5]. Behavioral changes include hyper-reactivity to novel environments, altered social interaction and increased aggression, and deficits in an array of cognitive tasks reflecting distinct cognitive domains affected by schizophrenia. These are accompanied by reduced neuronal plasticity, hyperfunction of mesolimbic and hypofunction of mesocortical dopaminergic systems, plus altered patterns of cortical gene expression which suggest disrupted GABAergic and glutamatergic neurotransmission [5, 6]. There is also evidence for reduced basal extracellular levels of glutamate in the hippocampus or prefrontal cortex [6, 7] and altered glutamate receptor antagonist or antipsychotic-evoked responses in the latter region [8–10].

The aim of the current research was to examine the ability of a 5-HT_6_ receptor antagonist, N-[3,5-dichloro-2-(methoxy)phenyl]-4-(methoxy)-3-(1-piperazinyl)benzene-sulfonamide (SB-399885), to reverse isolation-induced changes in a battery of cognitive tasks relevant to deficits in schizophrenia [11]; specifically a novel object discrimination (NOD) test of visual recognition memory, pre-pulse inhibition of the acoustic startle response (PPI) which assesses pre-attentional processing and sensorimotor gating, and a conditioned freezing response (CFR) paradigm which examines fear-motivated associative learning and memory. The 5-HT_6_ receptor is expressed in regions involved in learning and memory, including the cortex, hippocampus and amygdala, and selective antagonists elevate glutamate, acetylcholine and dopamine efflux in the rat prefrontal cortex and/or hippocampus. There is substantial preclinical evidence that they enhance rodent cognitive performance across a variety of domains and reverse NMDA receptor antagonist-induced deficits in pharmacological models of schizophrenia [12]. Although previous studies have shown reversal of isolation-induced deficits in visual recognition memory by 5-HT_6_ receptor antagonists [13–15] further evaluation against a broader array of cognitive deficits in this non-pharmacological neurodevelopmental model appears justified.

5-HT_6_ receptor activation stimulates cAMP accumulation and activates protein kinase A (PKA) via Gα_s_-coupling, but interactions with Gα_i/o_ and Gα_q_ [16] and links to ERK1/2 via Fyn-tyrosine kinase also occur [17–19]. Additional associations have been identified with Jun activation domain-binding protein-1 (Jab1) [20], mammalian target of rapamycin complex 1 (mTORC1) and several members of the mTOR pathway, as well as cyclin-dependent kinase 5 (Cdk5) [14] and multiple proteins regulating cytoskeleton dynamics [21, 22]. These interactions show enrichment in proteins implicated in learning and memory, and appear to underlie 5-HT_6_ receptor-mediated effects on cell survival, neuronal migration, neurite growth and dendritic spine morphogenesis [21, 23, 24]; processes impaired in neurodevelopmental disorders. To further characterize isolation-induced molecular changes and their potential reversal by SB-399885, this study employed a reverse-phase protein microarray technique [25] to examine hippocampal and frontal cortical expression and phosphorylation of selected intracellular signaling molecules (implicated in cell proliferation, differentiation, migration, apoptosis and cytoskeletal integrity, and which may modulate learning and memory), plus immunohistochemistry to examine Ki-67 as a marker of cell proliferation in the dentate gyrus.

Recent studies reveal decreased plasma IL-4, and increased IL-1 β, TNFα and IFNγ in isolation-reared rats [26–28]. Similar plasma [29] and prefrontal cortical [30] cytokine alterations are reported in schizophrenia, although plasma levels are influenced by antipsychotic treatment as well as the disease itself [31]. The potential pathophysiological involvement of alterations within the brain (where cytokines can act as neuromodulators as well as inflammatory mediators [32]) remains of interest and may represent a novel therapeutic target [33]. Animal studies are essential to investigate the roles of different environmental risk factors in the absence of confounding medication status and although most have focused on models involving direct immune activation it is interesting that isolation upregulates viral response genes in the hippocampus and frontal cortex despite the lack of direct immune challenge [6, 34]. Peripheral cytokine alterations in this model are reversed by atypical antipsychotics [27, 28] but possible brain regional cytokine alterations (and their potential drug reversal) have received less attention. The final aim of this study was therefore to use reverse-phase protein microarray to examine the impact of isolation rearing on hippocampal and frontal cortical expression of selected cytokines with differing pro- anti-inflammatory profiles (IL-1β, TNFα IL-6 and IL-10) and potential reversal of any changes by SB-399885; an interesting possibility given the 5-HT_6_ receptor is expressed in immune tissue [35].

SB-399885 is a potent brain penetrant 5-HT_6_ receptor antagonist with high affinity for the rat receptor (pKi = 8.81), 200-fold selectivity over all tested receptors, ion channels and enzymes, as well as a rapid onset (30 min) and long duration of action (15 h) following oral administration in the rat [36]. The number of animals and duration of testing makes it difficult to undertake a full dose-response analysis in group-housed and isolation-reared rats so, consistent with our previous evaluation of risperidone in this model [37], the current experiment examined two dose levels (5 and 10 mg/kg i.p.) in isolates and the highest dose only in group-housed controls. The pre-treatment time and choice of vehicle were selected for consistency with other studies in which a 10 mg/kg i.p. dose was behaviorally active without producing any motor impairment [38]. A 24-h post-injection time point was selected for tissue collection, as previous studies suggest this is appropriate for detection of prominent memory-related proteomic alterations lasting beyond drug occupancy of Gα_s_-coupled receptors [39].

## Materials and Methods

### Animals

**Fig. 1:**
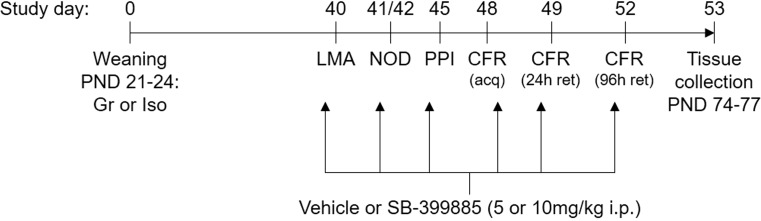
Summary of the experimental protocol. Fifty-five male Lister hooded rats were maintained in social groups of 3–4 (Gr) or isolation (Iso) from weaning on post-natal day (PND) 21–24. They received a total of six i.p. injections of vehicle (1% Tween 80 1 mL/kg), 5 or 10 mg/kg SB-399885 (lower dose in isolates only) over a 13-day period, on the days that locomotor activity (LMA), novel object discrimination (NOD), pre-pulse inhibition of acoustic startle (PPI), acquisition (acq) of a conditioned freezing response (CFR) and its retention (ret) 24 and 96 h later were examined. Behavioral tests were ordered from least to most aversive and treatments were administered 30 min prior to each, or immediately after CFR acquisition. Rats were killed 24 h after the final injection and brain tissue samples collected for reverse-phase protein microarray, western blotting and immunohistochemistry (*n* = 11 per housing-treatment combination)

55 male Lister hooded rats (Biomedical Services Unit, University of Nottingham, derived from Charles River stock) were weaned on post-natal day (PND) 21–24 and randomly assigned to group housing (*n* = 22; 3–4 per cage) or isolation (*n* = 33; 1 per cage), with allocation balanced according to litter of origin. Animals were housed in plastic cages (32 × 51 cm for groups and 25 × 42 cm for isolates) containing sawdust bedding but no environmental enrichment, and maintained in the same temperature (21 ± 2 °C) and humidity (55 ± 10%) controlled room on a 12-h light-dark cycle (lights on at 07.00 h) to allow visual, olfactory and auditory contact throughout the study. Handling was restricted to a singly weekly cage change and body weight measurement until the start of behavioral testing, which was conducted during the light phase (between 09.00 and 17.00 h). The doses and experimental design (Fig. 1) were chosen to comply with the three R’s of humane animal testing. All procedures were conducted in accordance with the Animals (Scientific Procedures) Act, 1986 and the ARRIVE guidelines [40], with approval of the University of Nottingham Local Ethical Committee.

### Drugs and Experimental Protocol

SB-399885 hydrochloride was purchased from Tocris Bioscience (Bristol, UK) and suspended in 1% aqueous solution of Tween 80 (Sigma-Aldrich, Dorset, UK) immediately prior to intraperitoneal (i.p.) administration in a volume of 1 mL/kg. Rats (*n* = 11 per group) received a total of six injections of vehicle, 5 or 10 mg/kg SB-399885 (lower dose in isolates only) over the course of 13 days (40–52 post-weaning), with a single injection on the days that locomotor activity, NOD, PPI and acquisition, retention and extinction of CFR were assessed, using a previously described test battery and methods with the tasks ordered from least to most aversive [37, 41]. Treatments were administered 30 min prior to each behavioral test, or immediately after CFR acquisition to preclude confounding drug effects on nociception [42] or affective state [38]. In all cases, the experimenter was unaware of the treatment received.

### Locomotor Activity

To determine the effect of SB-399885 on isolation-induced changes in spontaneous motor activity, ambulation and rears were assessed during a 1-h period in a novel arena on day 40 post-weaning. Thirty minutes after the first injection rats were placed in individual Perspex boxes (39 × 23.5 × 24.5 cm) with wire lids, surrounded by a photobeam activity system (San Diego instruments, CA, USA) consisting of eight parallel lower beams and four parallel upper beams. A single ambulation count was recorded for every two consecutive adjacent lower beam breaks, and a single rearing count recorded for every individual upper beam break.

### Novel Object Discrimination

To examine potential reversal of isolation-induced deficits in visual recognition memory by SB-399885, NOD was assessed on day 41 or 42 post-weaning, with a balanced mix of housing and treatment groups on each day. Twenty-six minutes after their second injection rats were returned to the same arena used for locomotor activity testing and sequentially received a further 3 min habituation to this arena in the absence of any objects, 1 min in the home cage, and two consecutive 3 min object exploration trials separated by a 2-h inter-trial interval in the home cage. In the first (familiarization) trial (starting 30 min post-injection), rats encountered two identical objects (cylindrical plastic bottles filled with water and covered in white masking tape, 8 cm high × 5 cm diameter) and for the second (choice) trial, one of these objects was randomly replaced with a novel object of the same size and shape but with four additional horizontal stripes of black electrical insulating tape. Exploration of each object, defined as sniffing, licking, chewing or having moving vibrissae while directing the nose towards and ≤ 1 cm from the object (but not sitting on an object in the absence of any directed interest), was recorded separately using stopwatches and used to calculate the choice trial discrimination ratio (exploration of the novel object/total choice trial object exploration).

### Pre-pulse Inhibition of Acoustic Startle

To evaluate the effect of isolation rearing and SB-399885 on sensorimotor gating, PPI was assessed on day 45 post-weaning. Thirty minutes after the third injection rats were placed in individual SR-Lab startle response chambers (San Diego instruments, CA, USA) and received 5 min acclimatization to background white noise (62 dB) then ten 120 dB (20 ms) startle pulse alone trials. These were followed by a further 50 startle trials, ten with no pre-pulse and the remainder preceded (by 100 ms) with a sub-threshold 72, 76, 80 or 84 dB (40 ms) pre-pulse. These were delivered in a pseudorandom order with a variable inter-trial interval of 10–20 s, and were followed by a final five startle alone trials. Individual whole-body startle responses were recorded for 100 ms from the onset of each startle pulse and used to calculate a total cumulative area under the curve (AUC). The mean percentage PPI for each trial type was calculated from the mean AUC for that trial type (after using a conditional statement to eliminate any extreme values greater than two standard deviations away from the mean, which can result from movement of the rat during startle delivery), using the equation % PPI = (pulse alone AUC – pre-pulse AUC)/pulse alone AUC × 100.

### Conditioned Freezing Response

To examine potential reversal of isolation-induced deficits in associative memory by SB-399885, acquisition, retention and extinction of CFR were assessed over a 5-day period. On day 48, post-weaning rats were placed into a two-compartment shuttle box with a light and dark side separated by an automated door and linked to shuttle box control and shocker units (Panlab SLab, Barcelona, Spain). After 30 s in the light side, the door opened and the latency for the animal to transfer to the dark side was recorded using a floor sensor which also triggered door closure. After 30 s in the dark side a 5-s conditioned stimulus (light and 3 kHz 89 dB tone) was delivered, paired with an unconditioned stimulus (1 s × 0.4 mA footshock delivered through the grid floor) during the last second. Rats received a total of three stimuli pairings separated by 55 s and the duration of freezing behavior (immobility, except for respiration, in a hunched posture with inactive vibrissae) following the first two pairings recorded separately using stopwatches. Immediately after the third pairing rats were removed from the apparatus and received the fourth injection of their allocated treatment. Twenty-four hours (day 49 post-weaning, 30 min after the fifth injection) and 96 h later (day 52 post-weaning, 30 min after the sixth injection), rats returned to the dark side for a total of 600 s, without any further footshocks. Freezing to the context was measured during the first 300 s, then the conditioned stimuli were presented for 5 s and freezing to cue was measured for the remainder of the trial.

### Tissue Collection

Rats were killed 24 h after the final injection (day 53 post-weaning) by concussion and immediate decapitation and the right hippocampus and frontal cortex dissected on a refrigerated table (4 °C) then frozen in liquid nitrogen (for protein microarray), while the left hemisphere was cryopreserved in 30% sucrose overnight at 4 °C then frozen in isopentane cooled by dry ice (for Ki-67 immunohistochemistry). Samples were stored at − 80 °C until analysis.

### Reverse-Phase Protein Microarray

Tissue samples were homogenized at 4 °C in lysis buffer (20 mM Tris, 1 mM EGTA, 320 mM sucrose, 0.1% Triton X100, 1 mM NaF, 10 mM B-glycerophosphate in distilled water adjusted to pH 7.6 with one EDTA-free protease inhibitor tablet per 10 mL (Roche Diagnostics, Newhaven, UK); 1 mL per 100 mg wet tissue weight) using a sonic probe (Soniprep 150: MSE Scientific Instruments, Crawley, UK; 10 s) and vertical rotator (Bibby Scientific, Stone, UK; 30 min, 4 °C), then centrifuged (3300*g*; Sigma 3-18 k: Newtown, UK; 5 min, 4 °C). Proteins in the supernatant were quantified by Lowry assay [43] and solubilized by addition of solubilization buffer (to achieve final concentrations of 4% SDS, 5% glycerol, 5% β-mercaptoethanol, 0.01% bromophenol blue and 0.0625 M Tris HCl), heated to 95 °C for 1 min, vortexed briefly, centrifuged (13,400*g*; Eppendorf 5417R: Stevenage, UK; 1 min) then adjusted to protein concentrations of 2 μg/μL. Samples were further diluted to 1.5 μg/μL using 4 × SDS buffer containing 10% β-mercaptoethanol and 5 units/mL benzonase) and the heat/vortex/centrifuge procedure repeated before spotting onto nitrocellulose-coated glass slides (Grace Bio-Labs, Bend, Oregon, USA) at 1.5, 0.75 and 0.375 μg/μL with a microarrayer (MicroGridII; Digilab, Marlborough, MA, USA). They were blocked overnight (4 °C with constant rocking) in 0.2% I-block (Tropix, Bedford, MA, USA), 0.1% Tween 20 in phosphate-buffered saline (TBST), then washed three times with TBST. Hippocampal slides were incubated overnight (4 °C with constant rocking) with one of 37 rabbit primary antibodies for proteins involved in PI3K/Akt, Erk, JNK, p38 MAP kinase, or Wnt/β-Catenin signaling, environmental stress and MAPK scaffolding (Cell Signaling Technology, Danvers, MA, USA; Table 1), or one of four goat primary antibodies for rat cytokines (R&D Systems, Minneapolis, MN, USA; Table 1), plus mouse β-actin primary antibody (1:1000; Sigma). Frontal cortical analysis was restricted to four JNK signaling intermediates which showed the greatest alteration in the hippocampus, plus the same four cytokines. Slides were washed, incubated in the dark for 30 min (room temperature with constant rocking) with 680 CW anti-mouse and 800 CW anti-rabbit infrared secondary antibodies (1:5000; LI-COR, Cambridge, UK), washed and then dried by centrifugation at 500×*g* for 5 min. Slides were scanned using a LI-COR Odyssey system (Cambridge, UK) and resultant TIFF images were processed with Axon Genepix Pro-6 Microarray Image Analysis software (Molecular Devices, Berkshire, UK) to obtain fluorescence data for each feature and generate gpr files. Protein signals were determined with background subtraction and normalized to β-actin expression using RPPanalyzer (German Cancer Research Centre, Heidelberg, Germany) [44]. Previous validation studies confirmed the selectivity of all primary antibodies, which detected single bands of the appropriate molecular weight on strip westerns [25 and unpublished observations]. It was not possible to measure all intermediates of the selected signaling pathways where suitable antibodies for microarray have yet to be validated, and quantification of these proteins was therefore beyond the scope of the current study.

**Table 1:** Primary antibodies used for reverse-phase protein microarray analysis

Pathway	Protein	Antibody dilution
Intracellular signaling proteins		
PI3K/Akt signaling	PI3K p85	1:500
	PI3K p110α	1:250
	PTEN	1:500
	p-PTEN (Ser380)	1:500
	p-PDK1 (Ser241)	1:1000
	p-Akt (Thr308)	1:50
	p-Akt (Ser473)	1:25
	p-Bad (Ser136)	1:250
Erk signaling	SHP-2	1:250
	c-Raf	1:500
	p-c-Raf (Ser259)	1:500
	MEK1 and MEK2	1:500
	Erk1/2	1:1000
	p-Erk1/2 (Thr202/Tyr204)	1:1000
JNK and/or p38 MAP kinase signaling	Rac1 and Cdc42	1:500
	p-MKK3 and p-MKK6 (Ser189/207)	1:250
	MKK4	1:250
	p-MKK4 (Thr261)	1:250
	MKK7	1:250
	TAK1	1:250
	p-TAK1 (Ser412)	1:250
	JNK	1:1000
	p-JNK (Thr183/Tyr185)	1:1000
	p38 MAPK	1:1000
	p-p38 MAPK (Thr180/Tyr182)	1:1000
	14–3-3ε	1:250
	p-HSP27 (Ser82)	1:50
	p-c-Jun (Ser63)	1:250
	p-ATF-2 (Thr71)	1:250
	p-Elk-1 (Ser383)	1:250
	STAT3	1:500
	p-STAT3 (Tyr705)	1:100
Wnt/β-catenin signaling	GSK-3β	1:500
	p-GSK-3β (Ser9)	1:500
	β-Catenin	1:250
Environmental stress	HSP90	1:1000
MAPK scaffolding	Flotillin-1	1:250
Cytokines		
	IL-1β	1:500
	IL-6	1:500
	IL-10	1:1000
	TNFα	1:1000

### Western Blotting

Significant changes in combined Rac1 and Cdc42 expression were detected by reverse-phase protein microarray but the antibody used was unable to differentiate between these proteins or their total versus phosphorylated states. Western blots were therefore performed using separate antibodies, both to validate that reverse-phase protein microarray data could be replicated with an alternative technique, and to provide further insight into altered Rho-GTPase expression and activation. Samples (2 μg protein/μl) were loaded (16 μg protein per lane) onto Mini-PROTEAN TGX gels (Bio-Rad, Watford, UK), separated (150 V for 1 h) then transferred to nitrocellulose membranes (100 V for 1 h at 4 °C) and blocked with a 5% milk solution (dried milk powder dissolved in Tris-buffered saline containing 5% Tween 20 (TBST); 1 h at room temperature). Portions containing proteins < 25 kDa were incubated overnight (at 4 °C) with rabbit polyclonal primary antibodies against Rac1 (1:500), p-Rac1 (Ser71, 1:500), Cdc42 (1:250; all Abcam, Cambridge, UK), or p-Cdc42 (Ser71, 1:150; Biorbyt Ltd., Cambridge, UK). Corresponding portions containing proteins > 25 kDa were incubated with rabbit polyclonal primary antibodies against the housekeeping protein GAPDH (1:20,000; Sigma-Aldrich, Dorset, UK). Membranes were washed three times with TBST, incubated in the dark for 1 h with 800 CW anti-rabbit infrared secondary antibodies (1:10,000; LI-COR, Cambridge, UK) then washed a further three times with TBST. Protein bands were detected and quantified using a LI-COR Odyssey system (Cambridge, UK) and data expressed as a percentage of GAPDH expression.

### Ki-67 Immunohistochemistry

Brains from 24 representative animals from group-housed and isolation-reared vehicle and 10 mg/kg SB-399885 treatment groups (*n* = 6 per group) were embedded in OCT (VWR International Ltd., Lutterworth, UK) at − 20 °C and serially sectioned in the coronal plane using a Leica CM 100 cryostat (Leica Microsystems, Knowlhill, UK). Sections were thaw-mounted onto 3-aminopropylmethoxysaline (APES)-coated slides and stored at − 20 °C until use. Ten sections from each rat, evenly spaced throughout the dentate gyrus, were chosen for Ki-67 immunohistochemistry and underwent the following protocol at room temperature in light-proof humidified chamber. Sections were fixed in 0.5% paraformaldehyde for 3 min and washed three times in PBS (5 min each), before 1 h incubation with monoclonal mouse Ki-67 primary antibody (1:100; Vector Laboratories, Peterborough, UK). Sections were washed, incubated for 1 h with Alexa Fluor 488 goat anti-mouse IgG3 secondary antibody (1:300; Invitrogen, Paisley, UK), washed again and counterstained with DAPI nuclear stain using Vectashield hard set mounting medium (Vector Laboratories, Peterborough, UK) then cover-slipped. Slides were stored at 4 °C and viewed at × 40 on a Nikon EFD-3 fluorescence microscope. The number of Ki-67 positive cells (discerned by co-localization of Ki-67 and DAPI staining) within the granular cell layer of the entire dentate gyrus and the sub-granular zone (defined as within a two cell layer of the granular cell layer on the hilar edge) were counted to provide a total value for the ten evenly spaced sections from each animal.

### Statistical Analysis

All analyses were performed using GraphPad Prism (v6.04) or IBM SPSS (v21) which can accommodate the unbalanced design caused by the lack of low dose SB-399885 evaluation in group-housed rats. Behavioral data were analyzed by three-way repeated measures ANOVA with time (locomotor activity), object (NOD), pre-pulse volume (PPI) or context/cue (CFR) as the within-subject factor and housing and treatment as between-subject factors. The NOD choice trial discrimination ratio, protein expression data and Ki-67 positive cell counts were analyzed by two-way ANOVA with housing and treatment as between-subject factors. ANOVAs were followed by Sidak post hoc tests with planned multiple comparisons (group-housed vehicle versus group-housed 10 mg/kg SB-399885, group-housed vehicle versus isolation-reared vehicle, isolation-reared vehicle versus isolation-reared 5 mg/kg SB-399885, isolation-reared vehicle versus isolation-reared 10 mg/kg SB-399885, and group-housed 10 mg/kg SB-399885 versus isolation-reared 10 mg/kg SB-399885) and *P* < 0.05 was regarded as statistically significant. Data are presented as the mean ± s.e.m. Visual representation of protein expression data in the form of a heat map was produced using Heatmapper software [45], and additional Pearson’s *r* correlation analyses were performed between signaling intermediates or cytokines significantly influenced by housing and/or treatment and locomotor activity (as indicated by total ambulatory activity in the first 35 min of the session), cognitive performance in NOD and CFR tasks (as indicated by the choice trial discrimination ratio and cued freezing 24 h post-acquisition, respectively) and Ki-67 positive cell counts.

## Results

### Locomotor Activity

**Fig. 2:**
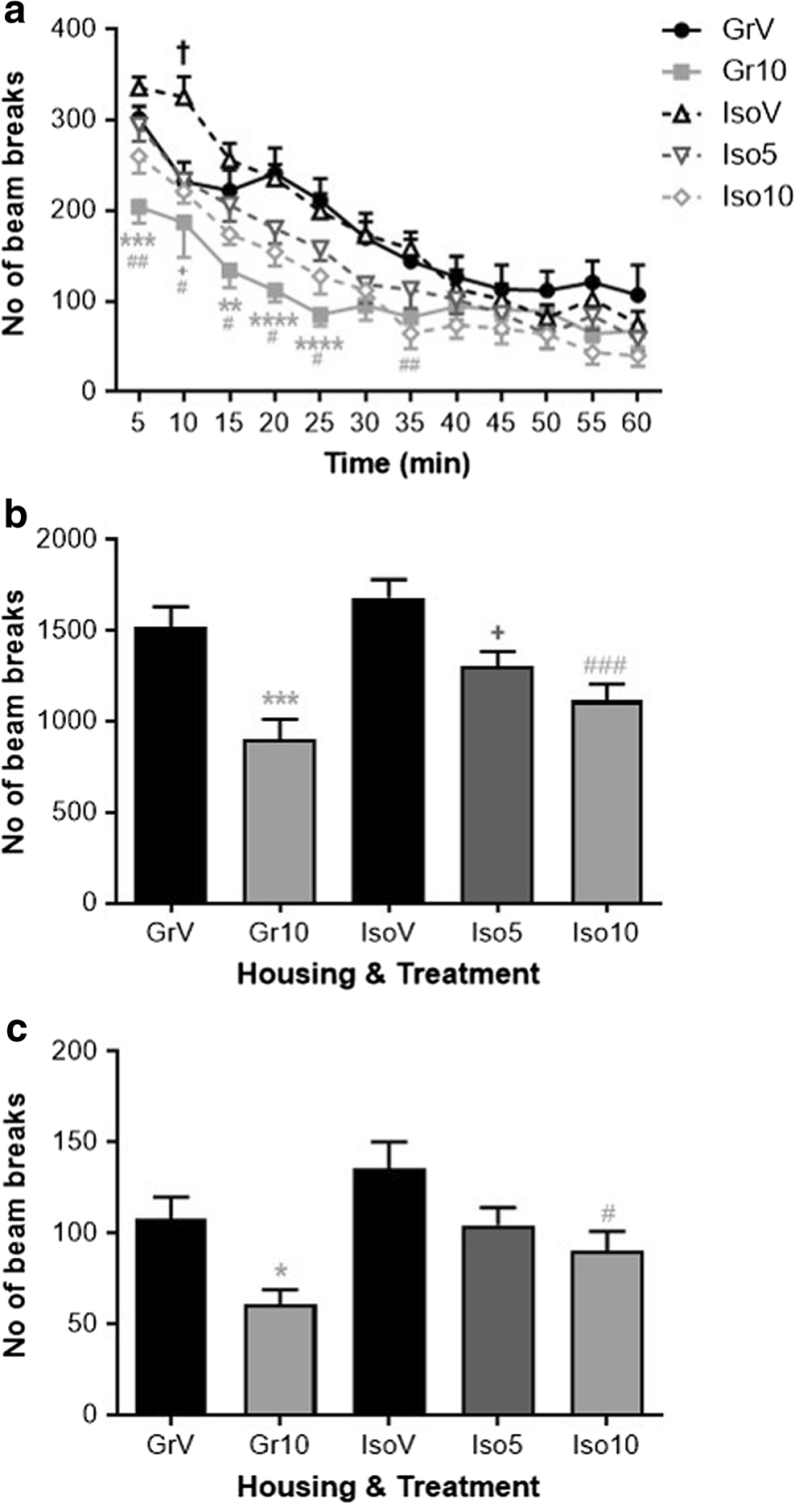
Effect of isolation rearing and SB-399885 on locomotor activity in a novel arena. Mean ± s.e.m. number of infrared beam breaks for **a** timecourse of ambulatory activity across the 1 h monitoring period and **b** total ambulation and **c** total rears within the first 35 min of the session. Group-housed (Gr) and isolation-reared (Iso) male Lister hooded rats received the first of six i.p. injections of vehicle (1% Tween 80 1 mL/kg), 5 or 10 mg/kg SB-399885 (lower dose in isolates only) 30 min prior to the start of the test session on day 40 post-weaning (*n* = 11 per group). **P < 0.01; ***P < 0.001; ****P < 0.0001 Gr10 versus GrV, ^+^*P* < 0.05 Iso5 versus IsoV, ^#^*P* < 0.05; ^##^*P* < 0.01 Iso10 versus IsoV, ^†^*P* < 0.05 IsoV versus GrV (three-way repeated measures ANOVA (**a**) or two-way ANOVA (**b**, **c**) with Sidak planned multiple post hoc comparisons)

Housing and treatment both influenced ambulatory activity during a 1-h period in a novel arena (time × housing *F*_(11,550)_ = 3.939, *P* ≤ 0.001, time × treatment *F*_(22,550)_ = 2.032, *P* ≤ 0.01). Ambulation was higher in vehicle-treated isolates than group-housed controls at the 10 min time point only (*P* < 0.05) but showed a prolonged decrease by SB-399885 irrespective of housing. Because all the observed between-group differences were in the first 35 min of the session (Fig. 2a), total activity counts during this period were also studied. Total ambulation was decreased by SB-399885 (*F*_(2,50)_ = 18.064, *P* ≤ 0.001; 5 mg/kg *P* < 0.05 and 10 mg/kg *P* < 0.001 versus relevant vehicle control), with the effect of housing just failing to reach significance (*F*_(1,50)_ = 3.516, *P* = 0.067) (Fig. 2b). Total rearing was decreased to a lesser extent by SB-399885 (*F*_(2,50)_ = 8.714, *P* ≤ 0.001; 10 mg/kg *P* < 0.05 versus vehicle) but in this case there was a main effect of housing (*F*_(1,50)_ = 4.169, *P* ≤ 0.05) (Fig. 2c).

### Novel Object Discrimination

**Fig. 3:**
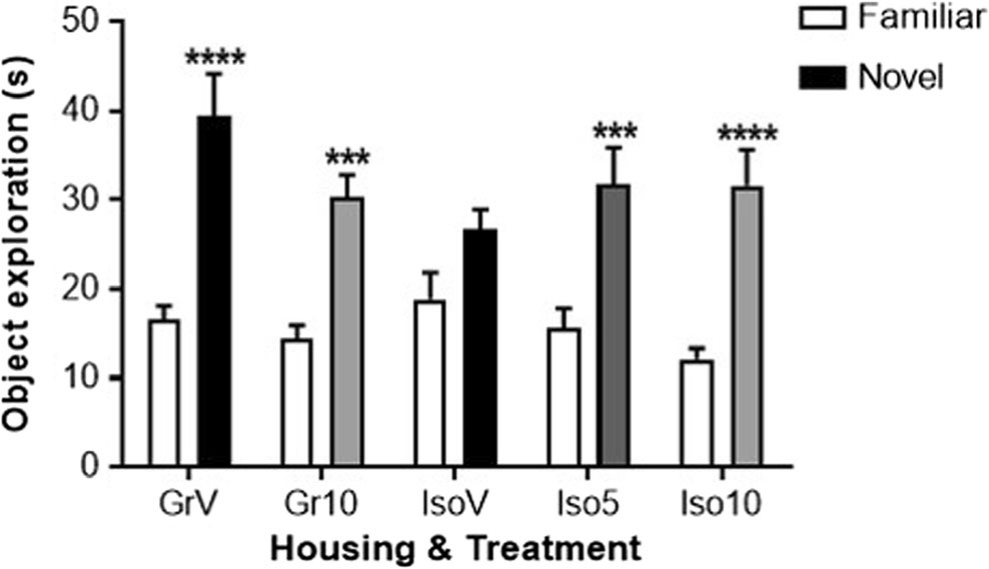
Effect of isolation rearing and SB-399885 on novel object discrimination following a 2-h inter-trial interval. Mean ± s.e.m. time (s) spent exploring the novel and familiar object during the choice trial. Group-housed (Gr) and isolation-reared (Iso) male Lister hooded rats received the second of six i.p. injections of vehicle (1% Tween 80 1 mL/kg), 5 or 10 mg/kg SB-399885 (lower dose in isolates only) 30 min prior to the start of the familiarization trial on day 41 or 42 post-weaning (*n* = 11 per group). ***P < 0.001; ****P < 0.0001 versus the familiar object in the same group of rats (three-way repeated measures ANOVA with Sidak post hoc test)

There were no spatial preferences for either identical object during the familiarization trial (data not shown), although the highest dose of SB-399885 decreased total levels of object exploration by isolation-reared rats (*P* < 0.01) in this trial alone (group-housed vehicle 49 ± 6 s, group-housed SB-399885 34 ± 4 s, isolation-reared vehicle 59 ± 5 s, isolation-reared 5 mg/kg SB-399885 45 ± 7 s, isolation-reared 10 mg/kg SB-399885 28 ± 6 s). In the choice trial (2 h later), there was a housing × treatment × object interaction (*F*_(1,50)_ = 5.907, *P* ≤ 0.05). Group-housed control animals were able to successfully discriminate the novel from the familiar object (*P* < 0.0001) and administration of SB-399885 to group-housed animals did not alter this. However, vehicle-treated isolates were unable to discriminate the novel object (*P* > 0.05) and this deficit was reversed by SB-399885 (*P* < 0.001 at 5 and *P* < 0.0001 at 10 mg/kg; Fig. 3). Importantly, this redistribution of object exploration occurred without any difference in total choice trial object exploration, and differences in cognitive performance during the choice trial cannot be explained by the pattern of altered object exploration during the familiarization trial or ‘learning phase’ of the task.

### Pre-pulse Inhibition of Acoustic Startle

Although all rats demonstrated the normal increase in percent PPI with increasing pre-pulse volume (*F*_(3,150)_ = 69.569, *P* ≤ 0.0001), this was not influenced by housing or treatment. There were no differences in basal startle reactivity or habituation to the startle pulse across the test session (data not shown).

### Conditioned Freezing Response

**Fig. 4:**
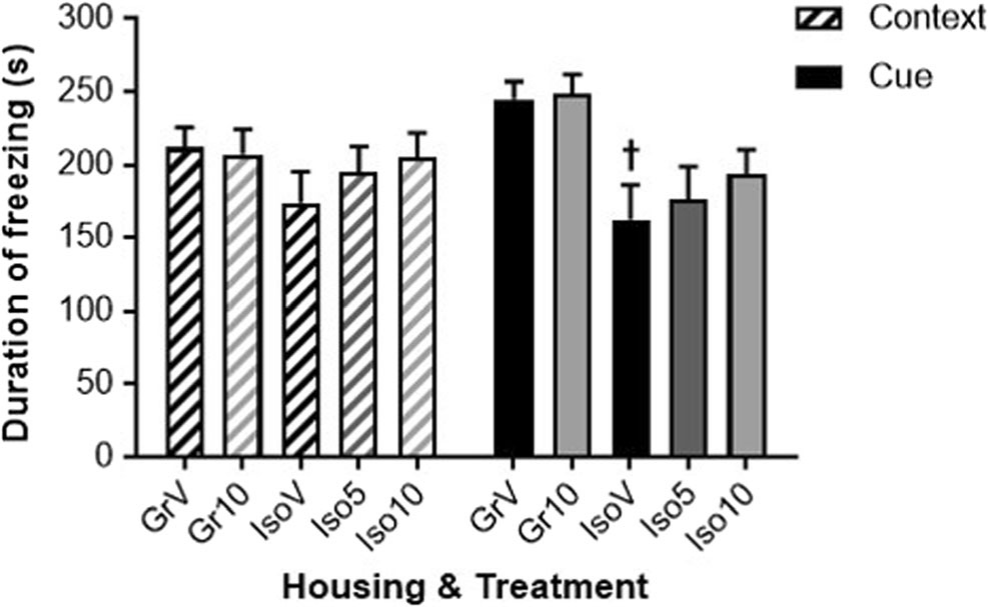
Effect of isolation rearing and SB-399885 on retention of a conditioned freezing response 24 h after the acquisition trial. Mean ± s.e.m. duration (s) of freezing to context in the 5 min period before, and freezing to cue in the 5 min period immediately after a 5-s tone and light presentation in the absence of further footshocks. Group-housed (Gr) and isolation-reared (Iso) male Lister hooded rats received the fifth of six i.p. injections of vehicle (1% Tween 80 1 mL/kg), 5 or 10 mg/kg SB-399885 (lower dose in isolates only) 30 min prior to the start of the test session on day 49 post-weaning (*n* = 11 per group). ^†^*P* < 0.05 versus the same treatment and freezing condition in group-housed rats (three-way repeated measures ANOVA with Sidak planned multiple post hoc comparisons)

There were no between-group differences in acquisition of the CFR (data not shown). On re-exposure to the apparatus 24 h later (in the absence of footshocks), there was a context/cue × housing interaction (*F*_(1,50)_ = 10.313, *P* ≤ 0.01), and while neither treatment or housing altered freezing to the context (Fig. 4), there was a main effect of housing on freezing to the cue (*F*_(1,50)_ = 13.626, *P* ≤ 0.001). Isolation rearing reduced this duration in vehicle-treated animals (*P* < 0.05 versus group-housed control) and although not fully reversed by SB-399885, post hoc comparison of each group to every other revealed the isolation-induced deficit was absent following drug treatment (*P* > 0.05; Fig. 4). On further return to the apparatus 96 h post-acquisition, there were reductions in freezing to context (*F*_(1,50)_ = 117.3, *P* ≤ 0.0001) and cue (*F*_(1,50)_ = 41.51, *P* ≤ 0.0001), consistent with extinction in the absence of further footshocks. At the 96 h time point, there was no context/cue × housing interaction nor any between-group differences in the level of extinction (data not shown).

### Reverse-Phase Protein Microarray and Western Blotting

**Fig. 5:**
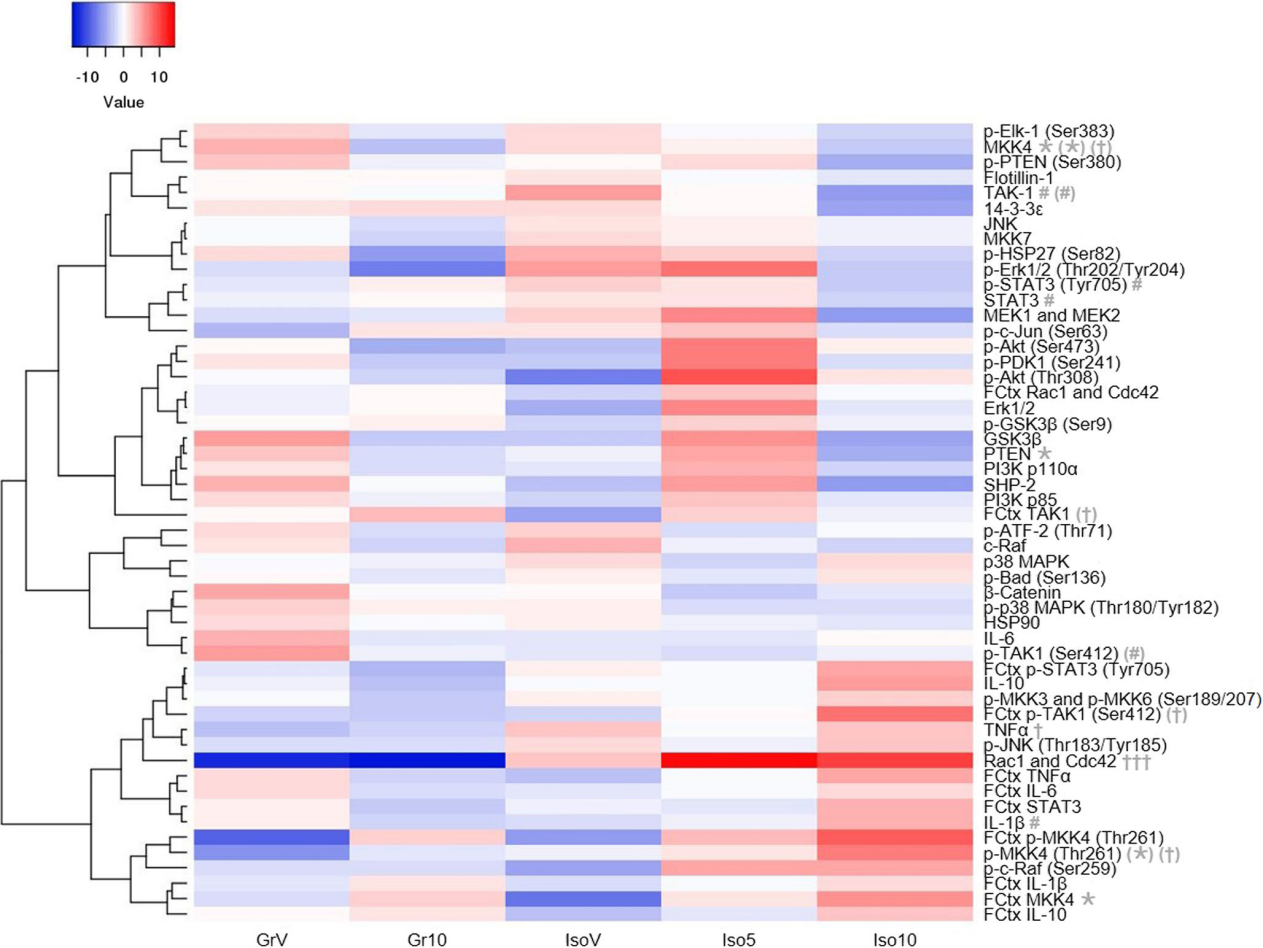
Effect of isolation rearing and SB-399885 on hippocampal and frontal cortical expression of signaling intermediates and cytokines. Blue (low) to red (high) signal intensity heat map representing the mean percentage difference in expression following each housing-treatment combination from the overall mean expression of that protein in the study as a whole; proteins are in the hippocampus unless prefixed by the frontal cortex (FCtx) abbreviation. Group-housed (Gr) and isolation-reared (Iso) male Lister hooded rats received a total of six i.p. injections of vehicle (1% Tween 80 1 mL/kg), 5 or 10 mg/kg SB-399885 (lower dose in isolates only) over a 13-day period, and were killed 24 h after the final injection (*n* = 11 per group). Statistical analyses were performed on the raw data (i.e., expression as a percentage of β-actin in the same individual), not differences from the overall study mean. ^†^*P* < 0.05 main effect of housing; *P < 0.05; ***P < 0.001 main effect of treatment; ^#^*P* < 0.05 housing × treatment interaction; symbols in parentheses indicate respective effects on the proportion in the phosphorylated state (two-way ANOVA; for significance with Sidak planned multiple post hoc comparisons see Fig. 6)

Visual representation in the form of a heat map revealed four broadly clustered groups of proteins (Fig. 5) whose general qualitative pattern appeared to be: similar or increased in isolates compared to group-housed controls, but decreased by the highest dose of SB-399885 (mainly components of JNK and/or p38 MAPK pathways); decreased by isolation, but increased by the lowest dose of SB-399885 (several members of the PI3K/Akt cascade); decreased by both does of SB-399885 (a small number of proteins from a range of pathways); similar or elevated in isolates compared to group-housed controls, and highest in isolates treated with the highest dose of SB-399885 (components of JNK and/or p38 MAPK pathways plus the majority of cytokines). Subsequent statistical analyses confirmed several significant changes, most of which were only apparent at the main effect level. In the hippocampus, these include effects of housing on TNFα (*F*_(1,50)_ = 4.300, *P* ≤ 0.05) and treatment on the negative PI3K/Akt regulator PTEN (*F*_(2,50)_ = 3.714, *P* ≤ 0.05), with similar trends for PI3K 110α, p-Akt (Thr308)) and Erk1/2 (*F*_(2,50)_ = 2.775, *P* = 0.073; *F*_(2,50)_ = 2.794, *P* = 0.071 and *F*_(2,50)_ = 2.639, *P* = 0.082 respectively), and a housing × treatment interaction for IL-1β (*F*_(1,50)_ = 4.120, *P* ≤ 0.05). There was also housing effect on the proportion of the multifunctional kinase TAK1 in the phosphorylated (Ser412) state within the frontal cortex (*F*_(1,50)_ = 5.086, *P* ≤ 0.05).

**Fig. 6:**
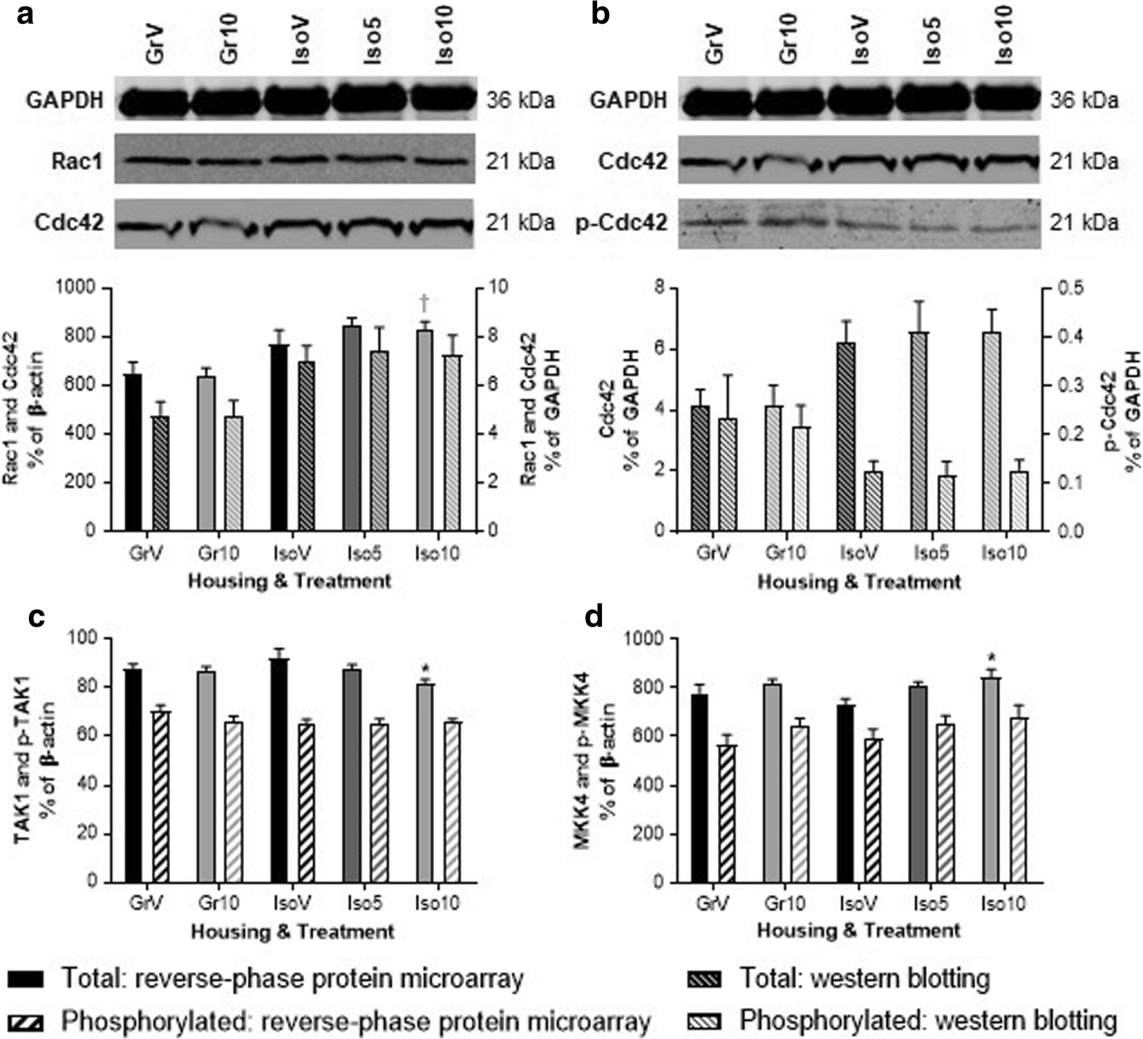
Effect of isolation rearing and SB-399885 on hippocampal and frontal cortical expression of selected JNK and/or p38 MAP kinase signaling intermediates. **a** Representative western blots, plus bar graph comparison of mean ± s.e.m. combined Rac1 and Cdc42 expression in the hippocampus using reverse-phase protein microarray and western blot analyses. Reverse-phase protein microarray data (solid bars) are expressed as a percentage of β-actin and shown on the left-hand *y*-axis, and western blot data (diagonal shaded bars) are expressed as a percentage of GAPDH and shown on the right-hand *y*-axis. **b** Representative blots and bar graph of mean ± s.e.m. hippocampal Cdc42 and p-Cdc42 (Ser71), quantified by western blotting and expressed as a percentage of GAPDH. Remaining bar graphs show reverse-phase protein microarray quantification of mean ± s.e.m. **c** Hippocampal TAK1 and p-TAK1 (Ser412) and **d** frontal cortical MKK4 and p-MKK4 (Thr261), all as percentages of β-actin. Group-housed (Gr) and isolation-reared (Iso) male Lister hooded rats received a total of six i.p. injections of vehicle (1% Tween 80 1 mL/kg), 5 or 10 mg/kg SB-399885 (lower dose in isolates only) over a 13-day period, and were killed 24 h after the final injection (*n* = 11 per group). *P < 0.05 versus vehicle under the same housing condition, ^†^*P* < 0.05 versus the same treatment in group-housed rats (two-way ANOVA with Sidak planned multiple post hoc comparisons)

More robust alterations were observed for a limited number of proteins associated with JNK and/or p38 MAP kinase signaling. These include an effect of housing on combined hippocampal levels of the Rho-GTPases Rac1 and Cdc42 (*F*_(1,50)_ = 12.561, *P* ≤ 0.001), which represented the greatest expression change in this study (Fig. 5). Values obtained by reverse-phase protein microarray were over 25% higher in isolates than group-reared rats following the highest dose of SB-399885 (*P* < 0.05; Fig. 6a). This housing effect was confirmed with different antibodies via the complimentary technique of western blotting (*F*_(1,50)_ = 9.688, *P* ≤ 0.01; Fig. 6a) and expression levels for individual animals showed a significant positive correlation between the two approaches (*P* = 0.0333). Greater selectivity of the western blot antibodies allowed attribution of the isolation-induced increase to total Cdc42 (*F*_(1,50)_ = 5.489, *P* ≤ 0.05; Fig. 6b), with decreases of over 40% in p-Cdc42 (*F*_(1,50)_ = 5.021, *P* ≤ 0.05; Fig. 6b) and over 70% in the proportion of cdc42 in the phosphorylated (Ser71) state (*F*_(1,50)_ = 9.497, *P* ≤ 0.01; Fig. 6b) while Rac1 remained unaffected. There was a housing × treatment interaction for hippocampal TAK1 (*F*_(1,50)_ = 4.578, *P* ≤ 0.05) which showed a small but significant decrease following administration of the highest dose of SB-399885 to isolation-reared rats (*P* ≤ 0.05 versus isolate vehicle; Fig. 6c), resulting in a further housing × treatment interaction for the proportion of TAK1 in the phosphorylated state (*F*_(1,50)_ = 5.476, *P* ≤ 0.05; Fig. 6c). SB-399885 had a main effect on total expression of the dual specificity kinase MKK4, which activates JNK and p38, and showed a small but significant increase (*F*_(2,50)_ = 3.770, *P* ≤ 0.05) of 16% in the frontal cortex following administration of the highest dose to isolation-reared rats (*P* ≤ 0.05 versus isolate vehicle; Fig. 6d). There were also effects of housing (*F*_(1,50)_ = 5.109, *P* ≤ 0.05) and treatment (*F*_(2,50)_ = 4.383, *P* ≤ 0.05) on the proportion of hippocampal MKK4 in the phosphorylated (Thr261) state, but the increase following administration of the highest dose to isolation-reared rats just missed the threshold for statistical significance (*P* = 0.0638; Fig. 5). Although at this time point hippocampal expression and phosphorylation of JNK itself appeared to be unaffected, there were housing × treatment interactions for expression (*F*_(1,50)_ = 4.358, *P* ≤ 0.05) and phosphorylation (Tyr705) (*F*_(1,50)_ = 6.249, *P* ≤ 0.05) of the downstream nuclear target STAT3 such that proportion in the phosphorylated state remained constant (Fig. 5).

### Ki-67 Immunohistochemistry

**Fig. 7:**
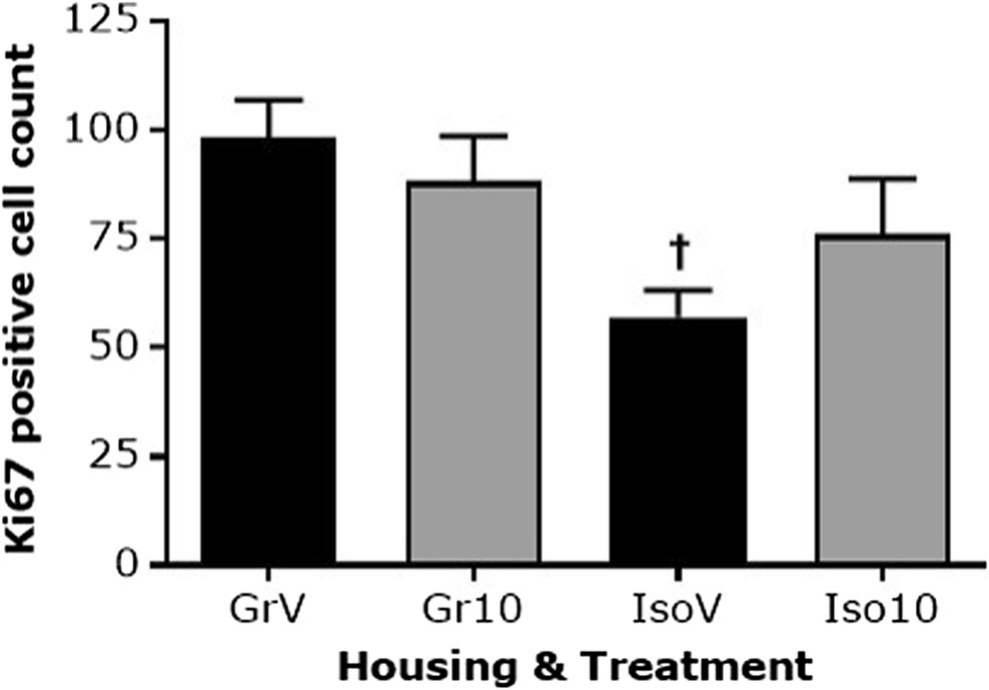
Effect of isolation rearing and SB-399885 on cell proliferation within the dentate gyrus. Mean ± s.e.m. number of Ki-67-positive cells in the granular cell layer and sub-granular zone, counted from ten evenly spaced sections per animal. Group-housed (Gr) and isolation-reared (Iso) male Lister hooded rats received a total of six i.p. injections of vehicle (1% Tween 80 1 mL/kg) or 10 mg/kg SB-399885 over a 13-day period, and were killed 24 h after the final injection (*n* = 6 per group). ^†^*P* < 0.05 versus the same treatment in group-housed rats (two-way ANOVA with Sidak planned multiple post hoc comparisons)

There was a main effect of housing on the number of Ki-67 positive cells in the dentate gyrus (*F*_(1,20)_ = 7.415, *P* ≤ 0.05). Isolation rearing decreased the Ki-67 positive cell count in vehicle-treated rats (*P* < 0.05 versus group-housed), and 10 mg/kg SB-399885 appeared to partially reverse this isolation-induced decrease since it was absent following drug treatment (*P* > 0.05 versus isolate vehicle but also *P* > 0.05 versus group-housed vehicle; Fig. 7).

### Correlation Analyses

**Fig. 8:**
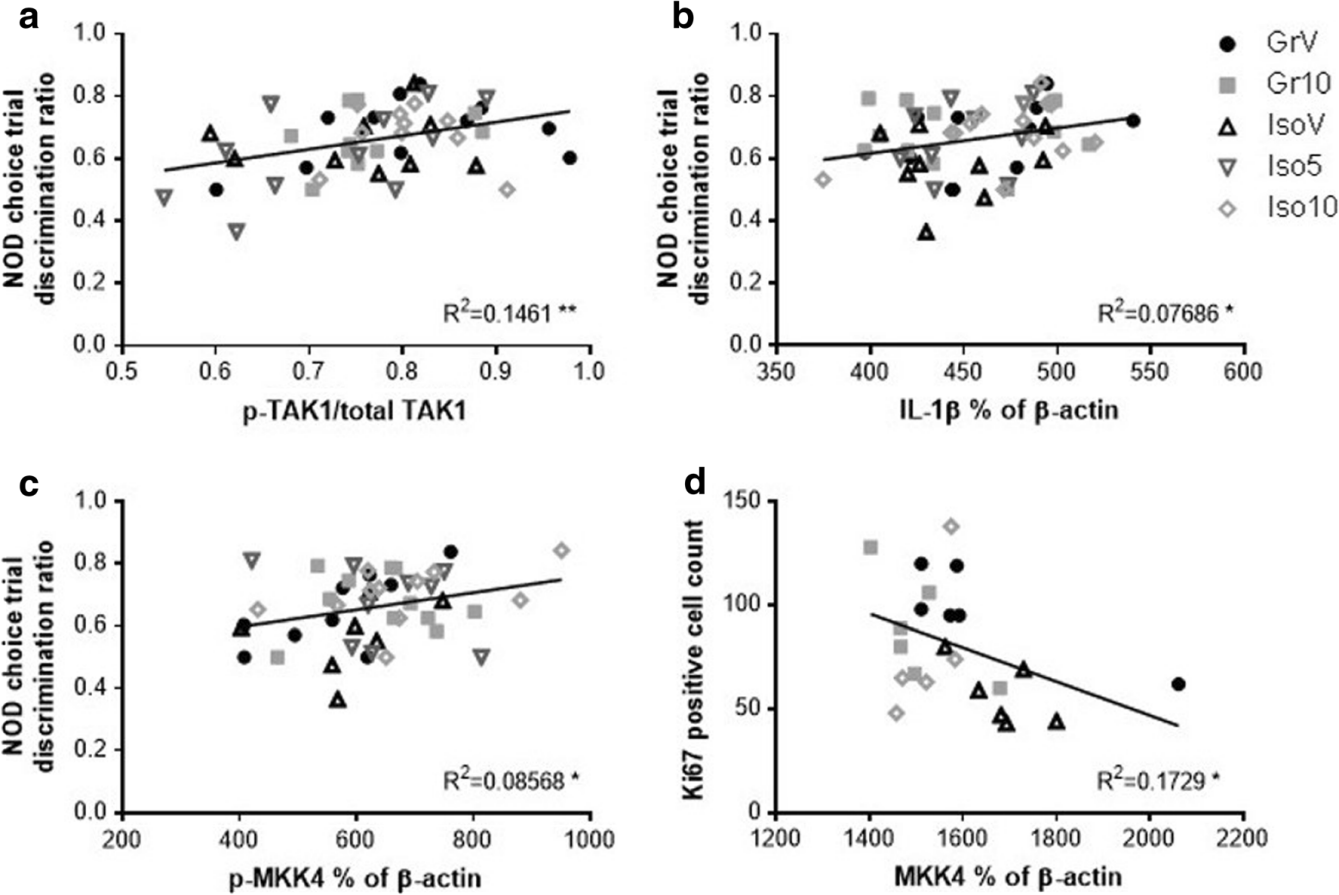
Correlations between hippocampal (**a**, **b**, **d**) and frontal cortical (**c**) expression of the signaling intermediates or cytokines significantly influenced by housing and/or treatment, and memory in the novel object discrimination task (**a–c**) or cell proliferation within the dentate gyrus (**d**). Each data point represents an individual animal; group-housed and isolation-reared male Lister hooded rats received a total of six i.p. injections of vehicle (1% Tween 80 1 mL/kg), 5 or 10 mg/kg SB-399885 (lower dose in isolates only) over a 13-day period, and were killed 24 h after the final injection (*n* = 11 per group, or *n* = 6 for Ki-67 expression following vehicle and the highest dose of SB-399885). *P < 0.05; **P < 0.01 (Pearson’s *r*)

Correlation analyses between behavioral performance or hippocampal cell proliferation and signaling intermediates or cytokines significantly influenced by housing and/or treatment revealed a negative association of hippocampal TAK1 with visual recognition memory in the NOD task, as indicated by the choice trial discrimination ratio (*P* = 0.0071; data not shown). There were positive relationships between NOD and hippocampal p-TAK1 (Ser412) as a proportion of total (*P* = 0.0056; Fig. 8a), hippocampal IL-1β (*P* = 0.0404; Fig. 8b) and frontal cortical p-MKK4 (Thr261) (*P* = 0.0459; Fig. 8c), together with a negative relationship between hippocampal MKK4 and Ki-67 positive cell count (*P* = 0.0485; Fig. 8d).

## Discussion

Post-weaning isolation of rats produces lasting neuroanatomical, neurochemical and behavioral alterations which emerge post-puberty, resemble some of the core features of schizophrenia, and offer the potential to detect reversal by novel agents operating on diverse pharmacological mechanisms. Although 5-HT_6_ receptor antagonists prolong memory in normal animals and reverse a variety of pharmacological deficits, their effects in relevant neurodevelopmental models are less well characterized. In addition to supporting known links to PI3K/Akt [14] and Erk1/2 [17–19] signaling and replicating reversal of isolation-induced recognition memory deficits [13, 15] with a structurally unrelated antagonist, this study provides the first evidence for potential modulation of associative memory and cell proliferation within the dentate gyrus in this model, and is also the first to reveal downstream effects of a 5-HT_6_ receptor antagonist on members of the JNK signaling pathway. The reverse-phase protein microarray technique provides a more direct functional measure than RNA analysis, and has previously been employed to study allergy [46], inflammation [47], cancer [48] and tumor necrosis factor receptor-associated periodic syndrome (TRAPS) [25, 49], although its current application to neurodevelopmental disorders and neuropharmacology is relatively novel.

5-HT_6_ receptor antagonists [50] including SB-399885 [38] can decrease locomotor activity and doses were therefore selected from the literature to avoid sedation. Previous studies [38, 51, 52] used Wistar rats and although some non-selective decrease in ambulation was observed here on first administration to Lister hooded rats (rather than the selective reversal of isolation-induced hyperactivity produced by an mGluR2/3 agonist [53]), it is important to emphasize that the subsequent effects of SB-399885 on cognition and Ki-67 expression cannot simply be attributed to locomotor confounds. In the NOD task, for example, isolation impaired choice trial discrimination without reducing total object exploration during the familiarization trial or ‘learning phase’ of the paradigm, and SB-399885 reversed this isolation-induced deficit despite having decreasing familiarization trial object exploration. Likewise any transient isolation-induced locomotor hyperactivity did not prevent expression of the CFR during acquisition training or contextual phases of the retention trials so would be an unlikely explanation for reduced cued freezing in these animals, and any potential sedative-type decrease in activity following SB-399885 would not confound scoring of freezing behavior with a hunched (rather than relaxed) body posture.

Locomotor hyperactivity in isolation-reared rats has been attributed to sensitization of the mesolimbic dopaminergic pathway [54–56] but the transient nature of this hyperactivity in the current study, together with the locomotor effect of SB-399885 in group-housed rats precludes any firm conclusions on drug-induced normalization of the housing effect. Likewise the lack of an isolation-induced PPI deficit in the current study (which may be related to strain and/or weaning age [57, 58]) prevented any evaluation of potential reversal by SB-399885, but other recent studies have just revealed that chronic 5-HT_6_ receptor antagonist administration can restore PPI in isolation-reared Wister rats [15] and may have some adjunctive benefit against the positive symptoms in female patient sub-groups [59].

5-HT_6_ receptor antagonists prolong visual recognition memory in normal animals and reverse NOD deficits in a variety of pharmacological models [12], and these effects are maintained following isolation rearing. NOD impairments in this model are reversed by compounds acting through diverse mechanisms, including the atypical antipsychotics clozapine and risperidone, an mGluR2/3 agonist, and antagonists of CB_1_, dopamine D_3_ and α2C adrenoceptors [28, 37, 53, 60–62]. Clinical evaluation of receptor-selective compounds on visual learning and memory in schizophrenia is still awaited, but modest effects of clozapine and risperidone [63, 64] appear to support the predictive validity of NOD in conjunction with the isolation rearing model. Glutamate hypofunction in the dorsal hippocampus is sufficient to impair NOD [65] so isolation-induced glutamatergic changes [7] including reduced VGLUT1 expression [66] may underlie this particular cognitive deficit. Unpublished microdialysis data from our laboratory reveal that isolation attenuates choice trial dopamine efflux from the prefrontal cortex; a key site for the NOD effects of D_3_ receptor antagonists [67] which increase dopamine efflux [68]. Elevation of hippocampal glutamate [69] and/or prefrontal cortical dopamine [70] efflux therefore represent plausible mechanisms via which 5-HT_6_ receptor antagonists could overcome isolation-induced NOD deficits, although further studies are required to determine whether these neurochemical effects are maintained following isolation. Interactions with mTOR signaling (which is disrupted in schizophrenia and by environmental factors [71]) appear relevant [14] and novel findings from the current study demonstrate hippocampal (but not frontal cortical) TAK1 expression and activation correlate with NOD, as do hippocampal IL-1β and frontal cortical p-MKK4. Although this does not necessarily prove a causal relationship recent studies show TAK1 expression is increased during acquisition, consolidation and retrieval of contextual fear memory [72], and implicated in the cognitive effects of GSK-3β inhibitors in a stroke model [73], while IL-1β can have positive effects on hippocampal-dependent memory [74, 75]. The potential association of hippocampal TAK1 and cytokines with visual recognition memory should be further investigated by intra-hippocampal administration of 5-HT_6_ receptor antagonists and selective modulators of TAK1 and cytokine signaling.

5-HT_6_ receptor antagonists have variable effects on pharmacological impairments in fear-motivated associative memory [76–78]. This first study to examine their effect on neurodevelopmental impairments in this cognitive domain appears to support some partial reversal worthy of further exploration, in that an isolation-induced deficit was only apparent in animals that received vehicle and not those treated with the 5-HT_6_ receptor antagonist. The locomotor effects of SB-399885 preclude systemic use of higher doses in an attempt to achieve full reversal of isolation-induced CFR deficits, but alternative (chronic rather than intermittent) dosing regimens in conjunction with antipsychotic agents would be valuable for assessment of potential adjunct efficacy. It is worth noting that to date the only compound to demonstrate full reversal of isolation-induced deficits in this task is donepezil [79], with risperidone and an mGluR2/3 agonist both inactive [37, 53]. SB-399885 is anxiolytic at lower doses than used here [38] and, consistent with this, actually decreases freezing behavior during contextual fear conditioning [80] perhaps by impairing consolidation in the dorsal hippocampus [81]. These effects are in direct opposition to any partial reinstatement of cued freezing in isolation-reared rats (following a different dosing schedule) and certainly suggest anxiolytic effects of SB-399885 are unlikely to confound current CFR data. Contextual fear memory (which was not modified by isolation rearing or SB-399885) is hippocampal dependent [82] and associated with short-term activation of JNK1 and c-Jun [83], sustained increases in PTEN and TAK1 expression, and a decrease in PI3K [72]. Cued fear memory is mediated by the amygdala [82] and this region therefore represents a key site for future investigation of 5-HT_6_ receptor antagonist-induced signaling alterations.

Expression of Ki-67 positive proliferating cells in the dentate gyrus granule cell layer is decreased in schizophrenia [84, 85] and although not a specific neuronal marker the localized decrease in known neurogenic regions supports a reduction in adult neurogenesis, which is in turn suggested to contribute to hippocampal and cognitive dysfunction in schizophrenia. Isolation rearing decreases cell proliferation and survival in the dentate gyrus [86] and the current decrease in Ki-67 further supports the face validity of the model. 5-HT_6_ receptor antagonists do not appear to enhance neurogenesis in normal adult rats [87] but the partial reversal of an isolation-induced deficit is intriguing and should be investigated in combination with antipsychotics, which share some effect on neurogenesis [88].

The most robust alterations in signaling protein expression in the current study were an isolation-induced increase in total hippocampal Cdc42 (but a decrease in activation), and region-specific effects of SB-399885 total TAK1 and MKK4 in isolation-reared rats. These were accompanied by more subtle changes in hippocampal TNFα (consistent with another recent isolation-induced increase [89]) and IL-1β (with the housing × treatment interaction consistent with a possible immune role of the 5-HT_6_ receptor [35]). The underlying mechanism for isolation-induced cytokine changes has yet to be fully elucidated, but both microglial activation [89] and the gut microbiome [90] may contribute. Attribution of specific behavioral or proliferation readouts to individual signaling alterations requires some caution. It is acknowledged that cell type or sub-region-specific effects [91, 92] may be masked by the current analysis of whole hippocampus or frontal cortex homogenates, and the full range of changes across dynamic signaling cascades may not be captured at a single time point, or by antibodies unable to differentiate MEK1 from MEK2, MKK3 from MKK6, and between JNK1–3 isoforms. Nevertheless, the current study has revealed several alterations worthy of further research, with an important stating point to determine whether these back translate from animal models to human post-mortem tissue. Cdc42 expression within the dorsolateral prefrontal and anterior cingulate cortices is decreased in schizophrenia [93–95], potentially due to single nucleotide polymorphisms [96], and although such changes have not yet been reported in the hippocampus or other brain regions the resulting alterations in cortical cytoskeleton dynamics may underlie spine deficits in layer 3 pyramidal cells [93–95]. Hippocampal Cdc42 activity is implicated throughout adult neurogenesis, from proliferation of neural progenitor cells, initial dendrite growth and arborization to spine maturation [97], and loss of Cdc42 leads to deficits in synaptic plasticity and memory recall [98]. The pattern of change in the current study did not appear to mirror isolation-induced deficits in cognition or Ki67 expression and their reversal (or partial reversal) by SB-399885 which is somewhat surprising given that 5-HT_6_ receptors promote neurite outgrowth in vitro via activation of Cdc42 [21], however as noted above a more dynamic insight may be obtained through study of multiple post-injection time points with a greater level of sub-region and cell-type specificity. At present specific dysfunction of neither TAK1 (which phosphorylates JNK via MKK4 and p38 via MKK3/6) nor MKK4 is implicated schizophrenia, but there is extensive evidence that downstream alterations in JNK signaling do contribute [99]. The current alterations in total TAK1 and MKK4 following six administrations of SB-399885 to isolation-reared rats over a 13-day period may be a compensatory adaptation to unexplored signaling changes at earlier time points, and although unlikely to have any immediate downstream consequence they do impact upon the pool available for subsequent phosphorylation. Cytokines and acute physiological stress produce rapid MKK4-JNK activation in the mouse hippocampus and cortex [100, 101] although to our knowledge the consequences of more prolonged stress for total protein levels have yet to be examined. It is interesting to note that animals with higher TAK1 and MKK4 expression (and thus potential for higher JNK activation) exhibited worse performance in the NOD task and lower numbers of Ki-67 positive proliferating cells in the dentate gyrus; a general picture consistent with the ability of JNK1 inhibition to increase neurogenesis [102]. Because TAK1 interacts directly with many protein products of schizophrenia risk genes without necessarily being altered itself, it has been proposed as a ‘hub’ within a dysfunctional network and a potentially attractive target via which to restore normal function [99], although this possibly has yet to be fully explored.

It is noteworthy that the two dose levels of SB-399885 appeared to exert opposing effects on some signaling intermediates. The 5-HT_6_ receptor is expressed on glutamatergic, GABAergic and to a lesser extent cholinergic neurons [103], and has been linked to multiple second messengers [14, 16–19], so it is plausible that 5-HT_6_ receptor ligands could exert diverging cell type-specific effects through separate messenger systems. Indeed there is accumulating evidence that both agonists and antagonists for this receptor exert similar behavioral and cognitive effects [104]. One speculation is that the effects of antagonists are mediated via blockade of 5-HT-induced GABAergic inhibition (with subsequent glutamatergic disinhibition), while agonists might activate a different population of 5-HT_6_ receptors located directly on glutamatergic neurones that normally receive little tonic 5-HT input and so might be relatively unaffected by an antagonist. An alternative possibility is that ligands acting as antagonists of one signaling pathway could act as agonists on another [105] presumably with different minimum effective doses. However even taking these proposals into consideration there may be a contradictory effect of SB-399885 on PI3K/Akt signaling, specifically on the balance between the negative regulator of this pathway, PTEN, and Akt activation. The main effect of treatment on PTEN and similar trend for p-Akt (Thr308) both appear due to small changes in the same direction (decreases in group-housed rats that received the high dose, and increases in isolates that received the low dose) rather than the normal pattern of opposition (only apparent in isolates that received the high dose). The paradoxical parallel effect, which we acknowledge did not reach robust statistical significance, may be partly resolved by the stronger influence of SB-399885 on p-Akt (Thr308) than p-PTEN (Ser380) such that any treatment effect favors Akt activation, but this appears inconsistent with the effect of 5-HT_6_ receptor antagonists on mTOR signaling [14, 106] and so requires future investigation.

In summary, the current study demonstrates the cognitive effects of a 5-HT_6_ receptor antagonist in normal animals are maintained following post-weaning social isolation. The available clinical data [107] suggest this neurodevelopmental model has good predictive validity for schizophrenia, and its continued use could therefore further elucidate the neurobiology and aid assessment of novel therapies for drug-resistant cognitive symptoms. However, at the single time point studied here, there was no change in hippocampal expression or phosphorylation of JNK or p38 themselves, nor of proteins such as 14-3-3ε, c-Jun, GSK-3β, β-Catenin and HSP90 which have been associated with schizophrenia and may represent additional treatment targets [92, 108–111]. Combination of isolation rearing with an additional developmental ‘hit’ (such as maternal immune activation or neonatal PCP administration [112, 113]) may induce a more complete spectrum of changes relevant to schizophrenia, including deficits in social cognition, and future work will evaluate the effect of 5-HT_6_ receptor antagonists and specific JNK pathway modulators in such models.
